# Ionic Liquid Confined in Mesoporous Polymer Membrane with Improved Stability for CO_2_/N_2_ Separation

**DOI:** 10.3390/nano7100299

**Published:** 2017-09-29

**Authors:** Ming Tan, Jingting Lu, Yang Zhang, Heqing Jiang

**Affiliations:** 1 Qingdao Key Laboratory of Functional Membrane Material and Membrane Technology, Qingdao Institute of Bioenergy and Bioprocess Technology, Chinese Academy of Sciences, Qingdao 266101, China; tanming@qibebt.ac.cn (M.T.); lujt@qibebt.ac.cn (J.L.); zhangyang@qibebt.ac.cn (Y.Z.); 2 University of Chinese Academy of Sciences, Beijing 100049, China; 3 Institute of Coal Chemistry, Chinese Academy of Science, Taiyuan 030001, China

**Keywords:** gas separation, supported membrane, mesoporous polymer, ionic liquid, high stability

## Abstract

Supported ionic liquid membranes (SILMs) have a promising prospect of application in flue gas separation, owing to its high permeability and selectivity of CO_2_. However, existing SILMs have the disadvantage of poor stability due to the loss of ionic liquid from the large pores of the macroporous support. In this study, a novel SILM with high stability was developed by confining ionic liquid in a mesoporous polymer membrane. First, a mesoporous polymer membrane derived from a soluble, low-molecular-weight phenolic resin precursor was deposited on a porous Al_2_O_3_ support, and then 1-ethyl-3-methylimidazolium tetrafluoroborate ([emim][BF_4_]) was immobilized inside mesopores of phenolic resin, forming the SILM under vacuum. Effects of trans-membrane pressure difference on the SILM separation performance were investigated by measuring the permeances of CO_2_ and N_2_. The SILM exhibits a high ideal CO_2_/N_2_ selectivity of 40, and an actual selectivity of approximately 25 in a mixed gas (50% CO_2_ and 50% N_2_) at a trans-membrane pressure difference of 2.5 bar. Compared to [emim][BF_4_] supported by polyethersulfone membrane with a pore size of around 0.45 μm, the [emim][BF_4_] confined in a mesoporous polymer membrane exhibits an improved stability, and its separation performance remained stable for 40 h under a trans-membrane pressure difference of 1.5 bar in a mixed gas before the measurement was intentionally stopped.

## 1. Introduction

Membrane-related processes including gas separation membranes and supported ionic liquid membranes (SILMs) are considered to be very promising technologies. Owing to their high permeability and selectivity of CO_2_, SILMs have a promising prospect of application in biogas purification and flue gas separation [1,2,3]. Usually, SILM supports are microfiltration membranes made from polyethersulphone (PES) [3,4,5,6,7], polyvinylidene fluoride (PVDF) [7,8,9,10,11], polypropylene (PP) [7,12], nylon [6,13], ceramics [14,15,16], and their pore sizes are in the range from 0.1 to 0.45 um. For example, Adibi et al. prepared an SILM by impregnating alumina porous supports in ionic liquid [1,3-di(3-methyl-imidazolium) propane bis(trifluoromethylsulfonyl) imide], for which the ideal CO_2_/CH_4_ selectivity was 27.1 at 27 °C [16]. However, the application of SILMs in industry is still limited because ionic liquid in this kind of membrane is mainly immobilized in the support by capillary forces, and can easily leak out from the larger pores of the support [6,7,17], whose pore size distribution is wide under a high trans-membrane pressure difference. For instance, Neves et al. observed that the membrane weight of an SILM in a hydrophilic support decreased continuously at a trans-membrane pressure difference of 1 bar [18]. Albo et al. introduced four kinds of ionic liquid with phosphonium cation into either hydrophilic or hydrophobic PVDF and found that the membrane weight loss did not reach a stable value at a 1 bar pressure difference [19]. 

According to the Young-Laplace equation, the maximum trans-membrane pressure difference that SILMs can withstand is relevant to the pore size of the support membrane. Decreasing the pore size will increase capillary forces, thereby enhancing the stability of an SILM. Tsuru et al. immobilized a high CO_2_ solubility ionic liquid (IL) [emim][Ac] into mesoporous TiO_2_ with an average pore size of approximately 20 nm [20] and nanoporous TiO_2_ and SiO_2_–ZrO_2_ layers with average pore sizes of approximately 2.5 and 1 nm, respectively [21]. The stability of the membranes was ensured for 25 h at an applied pressure of 4 bar in the separation of CO_2_ and N_2_. Obviously, a narrow pore size distribution of the support will clearly have a positive effect on the stability of an SILM [22,23]. 

In the past decades, significant efforts have been devoted to developing mesoporous polymers with large surface areas and very narrow pore size distributions. The evaporation induced self-assembly (EISA) method was widely used to obtain mesoporous polymers [24,25,26]. Mesoporous phenolic resin with different structures and uniform pore size can be generated by simply adjusting the ratio of the phenol/template and the calcination temperature [25]. 

Obviously, a mesoporous polymer with uniform and smaller pore sizes is quite suitable to use as the support of an SILM. In this study, ionic liquid 1-ethyl-3-methylimidazolium tetrafluoroborate [emim][BF_4_] confined in a mesoporous polymer membrane with high stability was developed. A mesoporous polymer membrane was deposited on a porous Al_2_O_3_ support by self-assembly of triblock copolymers with soluble, low-molecular-weight phenolic resin precursors, followed by high-temperature treatment under Ar flow. The ionic liquid [emim][BF_4_] was immobilized in the mesoporous polymer membrane, forming a novel SILM. Effects of trans-membrane pressure difference on SILM separation performances were investigated. By confining ionic liquid into the mesopores of the polymer, an improved stability was achieved.

## 2. Results and Discussion

**Figure 1:**
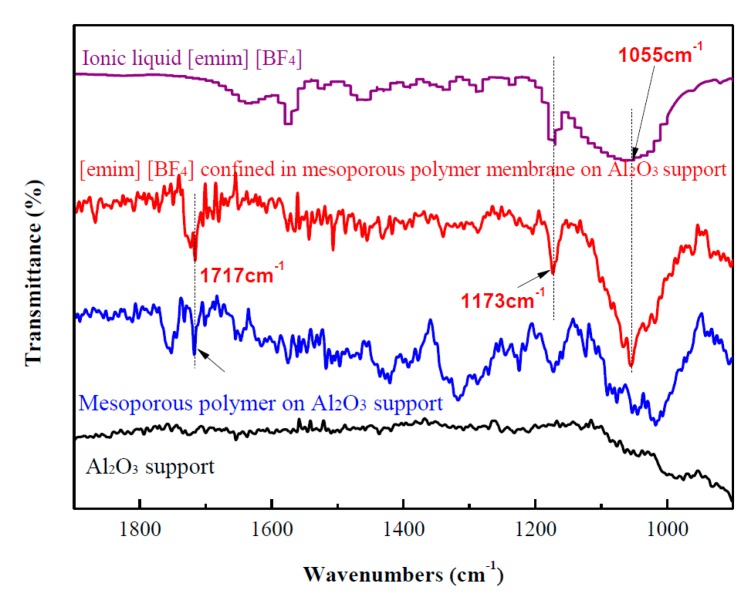
Fourier-transform infrared (FTIR) spectra of Al_2_O_3_, mesoporous polymer membrane on Al_2_O_3_ support, [emim][BF_4_] confined in mesoporous polymer membrane on Al_2_O_3_ support, and pure [emim][BF_4_].

Figure 1 presents the Fourier-transform infrared (FTIR) spectra of an Al_2_O_3_ support, a mesoporous polymer membrane on the Al_2_O_3_ support, [emim][BF_4_] confined in the mesoporous polymer membrane on the Al_2_O_3_ support, and pure [emim][BF_4_]. Compared with the Al_2_O_3_ support, the spectrum of the mesoporous polymer membrane on the Al_2_O_3_ support exhibits a strong band at 1717 cm^−1^ caused by the vibration of the tetra-substituted benzene ring [25], indicating that mesoporous polymer phenolic resin was successfully deposited on the surface of the Al_2_O_3_ support. The FTIR spectrum of [emim][BF_4_] confined in the mesoporous polymer membrane on the Al_2_O_3_ support shows a strong band at 1055 cm^−1^ arising from B–F stretching in BF_4_^−^ and another band at 1173 cm^−1^ arising from the in-plane C–H deformation vibration of the imidazole ring [27], suggesting that ionic liquid [emim][BF_4_] was immobilized in the mesopores of the polymer phenolic resin. Based on these results, it can be seen that [emim][BF_4_] confined in the mesoporous polymer membrane was successfully deposited on the Al_2_O_3_ support, which can be further confirmed by SEM and energy-dispersive X-ray spectroscopy (EDXS) measurements.

**Figure 2:**
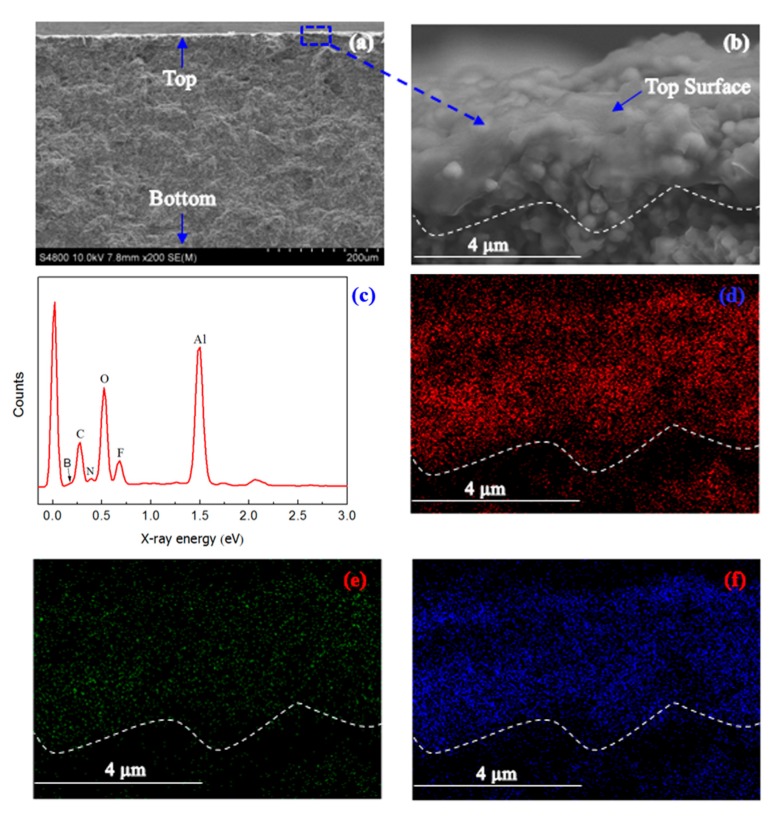
(**a**) SEM image, (**b**) Partially magnified SEM image, (**c**) Total element mapping, (**d**) Element C mapping, (**e**) Element B mapping and (**f**) Element F mapping of [emim][BF_4_] confined in mesoporous polymer membrane on Al_2_O_3_ support.

Figure 2a,b show the cross-sectional SEM images of [emim][BF_4_] confined in the mesoporous polymer membrane on the Al_2_O_3_ support. The main part of the membrane is composed of a coarse alumina layer with large pores. On the top part of this membrane is a separation layer, consisting of phenolic resin and ionic liquid [emim][BF_4_] with a thickness of around 500 nm. EDXS was used to analyze the composition of the upper layer (Figure 2c). The characteristic peaks attributed to elements C (Figure 2d), B (Figure 2e), and F (Figure 2f) were found in the survey spectrum. From the corresponding elemental mapping, it can be seen that C, B, and F were mainly distributed on the top of the membrane, as indicated by the white dash line. According to SEM and elemental mapping images, it can be concluded that [emim][BF_4_] confined in the mesoporous polymer membrane was successfully grown on the Al_2_O_3_ support. 

**Figure 3:**
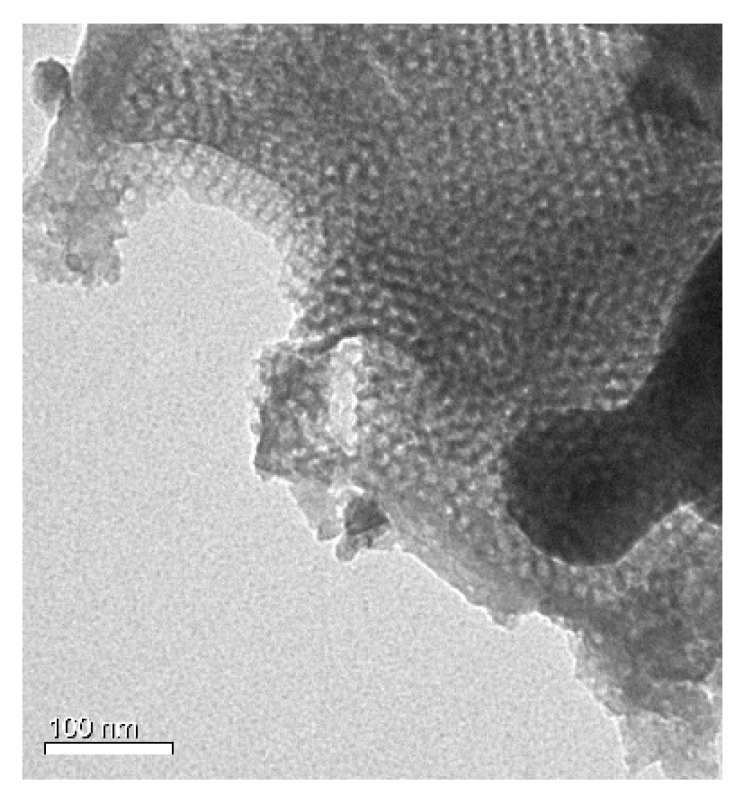
Transmission electron micro (TEM) image of mesoporous phenolic resin scratched from Al_2_O_3_ support.

**Figure 4:**
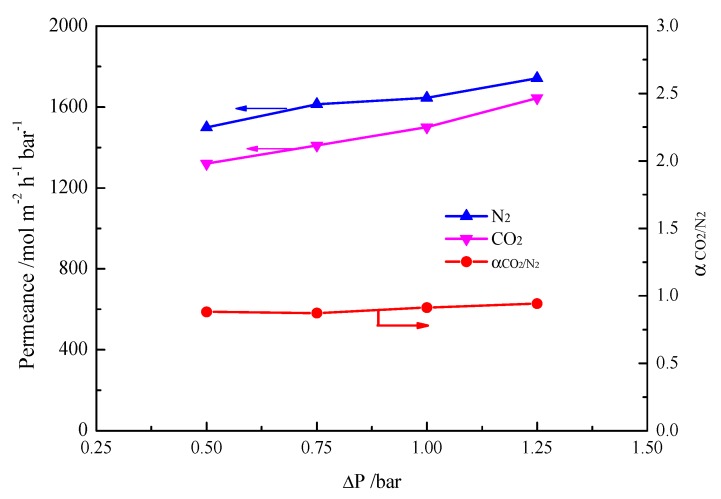
Effect of trans-membrane pressure difference (Δ*P*) on permeances and CO_2_/N_2_ ideal selectivities of mesoporous polymer membrane on Al_2_O_3_ support.

In order to observe the pore size of the phenolic resin derived from the resol precursor, the polymer membrane was scratched from the surface of the alumina support before introducing ionic liquid. Transmission electron micro (TEM) was used to observe the pore size of the phenolic resin. It can be seen from Figure 3 that the pore size is around 7 nm, which is in accordance with the previous reports [25]. Thus, the transport of gases through such a porous membrane should follow the mechanism of Knudsen diffusion. Figure 4 shows the permeances and CO_2_/N_2_ ideal selectivities of the mesoporous polymer membrane on the Al_2_O_3_ support. At a trans-membrane pressure difference of 1 bar, the CO_2_ permeance is as high as 1500 mol/m^2^·h·bar, and the CO_2_/N_2_ selectivity is only 0.9, which is quite close to the theoretical Knudsen separation factor of 0.8. Obviously, the phenolic resin-based membrane with a pore size of around 7 nm could not well separate the CO_2_ from the gas mixture of CO_2_/N_2_. 

**Figure 5:**
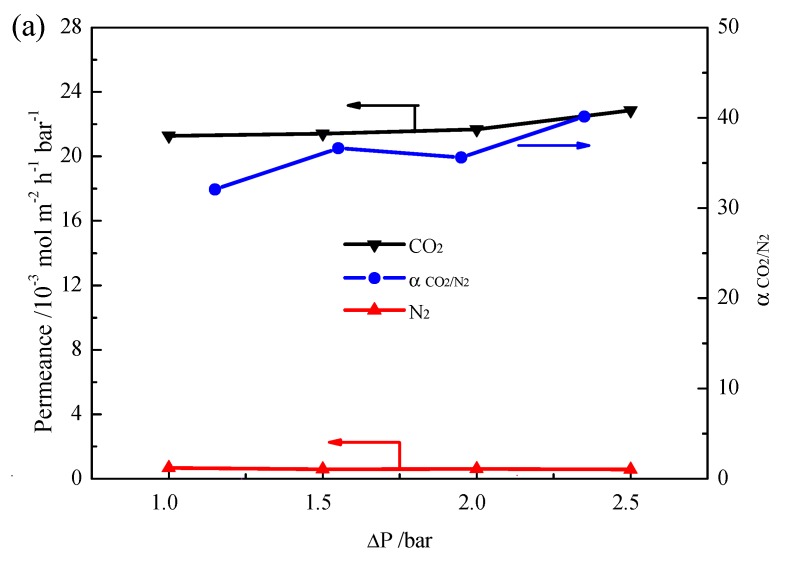

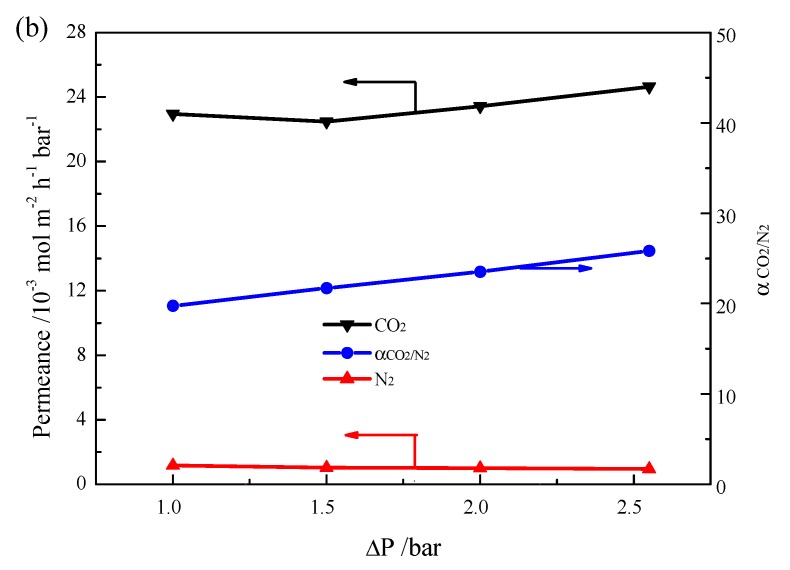
Effect of trans-membrane pressure difference (Δ*P*) on permeances and selectivities of [emim][BF_4_] confined in mesoporous polymer membrane on Al_2_O_3_ support at 25 °C. (**a**) CO_2_/N_2_ ideal selectivity; (**b**) CO_2_/N_2_ actual selectivity, feed concentration CO_2_/N_2_ = 50/50% (*v*/*v*).

After introducing ionic liquid into the pores of the phenolic resin, the pores will be full of ionic liquid under vacuum, forming a novel SILM, [emim][BF_4_] confined in the mesoporous polymer membrane on the Al_2_O_3_ support. The permeation of gases through the novel SILM follows the solution-diffusion mechanism. Figure 5a presents the gas permeance and ideal selectivity of the novel SILM for pure CO_2_ or N_2_ gas at different trans-membrane pressures. The permeance of CO_2_ is significantly higher than that of N_2_, and the ideal selectivities of CO_2_/N_2_ based on the permeation tests are in the range of 32–40. When the trans-membrane pressure difference is 2.5 bar, the ideal selectivity of CO_2_/N_2_ is about 40. High permeability of CO_2_ should result from its high solubility and diffusivity in ionic liquid. N-containing organic heterocyclic was reported to play an important role in interacting with CO_2_, thus increasing its solubility [28]. Kazarian et al. found that CO_2_ could also interact with BF_4_^−^ owing to a Lewis acid/base interaction, and thus facilitate CO_2_ transport [29]. Lim et al. pointed out that CO_2_ molecules can easily squeeze into the inter-ion space between the cation and anion [30]. Blanchard et al. [31] and Shim et al. [30] further explained that this behavior is due to the existence of “void space” or “free volume” in the ionic liquid. Figure 5b presents the gas permeance and actual selectivity of the novel SILM for mixed CO_2_/N_2_ gas (50% CO_2_ and 50% N_2_) at different trans-membrane pressures. The actual selectivities of CO_2_/N_2_ based on the permeation tests are in the range of 23–25. When the trans-membrane pressure difference is 2.55 bar, the actual selectivity for mixed CO_2_/N_2_ is about 25. Obviously, the ideal selectivity is much higher than the actual selectivity of CO_2_/N_2_; this is because the actual selectivity for mixed CO_2_/N_2_ gas is related to the ideal selectivity of CO_2_/N_2_ and the pressure of the permeate side and the feed side [3,32]. The higher the ideal selectivity and the ratio of the pressure in the feed side to the permeate side are, the closer the ideal selectivity and the actual selectivity will be [3,32]. As shown in Figure 5, the CO_2_/N_2_ selectivity increased slightly with the increase of the trans-membrane pressure difference gradient across the membrane. It is necessary to point out that the novel SILM shows a high CO_2_/N_2_ selectivity at the high trans-membrane pressure difference of 2.55 bar, and the permeation flux of N_2_ is still at a very low level, indicating the good stability of the membrane. The ideal CO_2_/N_2_ selectivity of the novel SILM is comparable with other SILMs [20,21,33] and found to be very close to the intrinsic value of the [emim]BF_4_] (ideal selectivity: 44 [3]) using a lag-time technique [34], although the permeance of the mixed gas for the novel SILM is lower than that reported in the literature [3]. 

**Figure 6:**
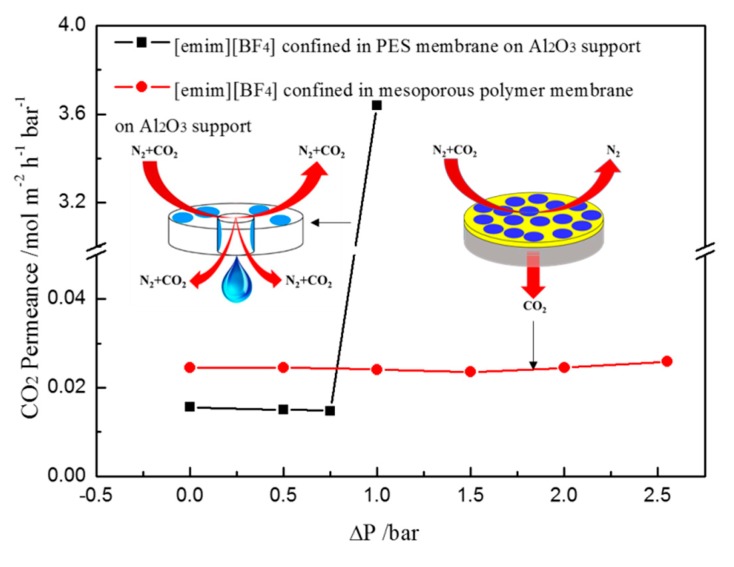
Stability comparison between [emim][BF_4_] confined in mesoporous polymer membrane on Al_2_O_3_ support and [emim][BF_4_] confined in polyethersulphone (PES) macroporous membrane on Al_2_O_3_ support.

For comparison, the ionic liquid [emim][BF_4_] was introduced into the porous PES membrane with a mean pore size of around 0.45 μm. Figure 6 presents the CO_2_ permeance of [emim][BF_4_] confined in the PES membrane and mesoporous polymer membrane at different trans-membrane pressure differences using the mixed gas (50% CO_2_ and 50% N_2_) as feed gas. The CO_2_ permeance increases suddenly from 0.015 to 3.59 mol/m^2^·h·bar as the trans-membrane pressure difference increases from 0.75 to 1 bar, which might be caused by the leakage of [emim][BF4] from the pores of the PES membrane. This indicates that the ionic liquid [emim][BF_4_] supported by the PES membrane with a pore size of around 0.45 μm cannot tolerant the trans-membrane pressure difference of more than 1 bar. In contrast, the CO_2_ permeances almost remain constant at about 0.024 mol/m^2^·h·bar when the trans-membrane pressure difference increases from 0 to 2.5 bar, indicating the better stability of the novel SILM. It can be seen from Figure 6 that the CO_2_ permeance of the novel SILM is larger than that of the ionic liquid [emim][BF_4_] supported by the PES membrane, which is probably caused by the difference of the thickness of the IL layer between the two kinds of membranes.

**Figure 7:**
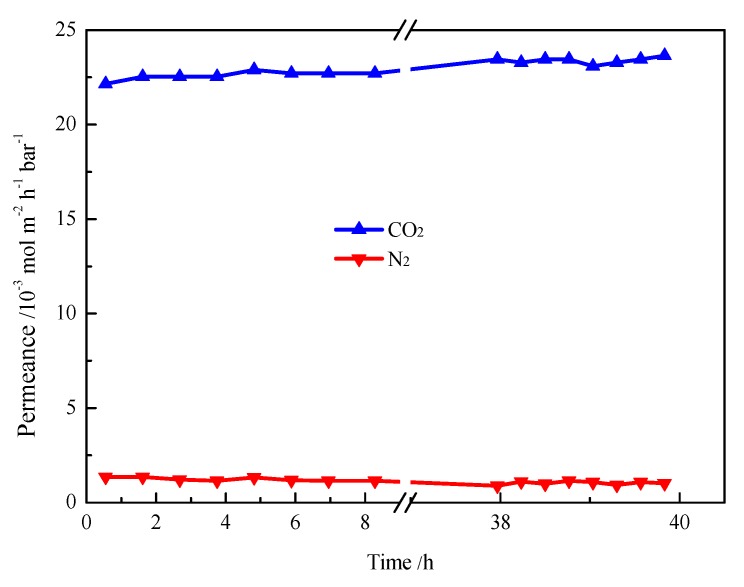
CO_2_ and N_2_ separation stability of [emim][BF_4_] confined in mesoporous polymer membrane on Al_2_O_3_ support, Δ*P* = 1.5 bar, feed concentration CO_2_/N_2_ = 50/50% (*v*/*v*).

In addition, permeation tests with binary gas mixtures (CO_2_/N_2_) were carried out to assess the long-term stability of the novel SILM. As shown in Figure 7, under the trans-membrane pressure of 1.5 bar, the permeances of CO_2_ and N_2_ almost keep constant for 40 h, indicating that the membrane can withstand a high trans-membrane pressure difference without performance degradation. 

## 3. Materials and Methods 

### 3.1. Materials 

The ionic liquid, 1-ethyl-3-methylimidazolium tetrafluoroborate ([emim][BF_4_]), was purchased from J&K Scientific Ltd. (Beijing, China) with a mole fraction purity of 99%. Alumina powders were obtained from Xuan Cheng Jing Rui New Material Co., Ltd. (Xuancheng, China) with an average particle size of 100 nm. Polyethersulfon (PES) membranes with a mean pore diameter of 0.45 μm were bought from Hangzhou Anow microfiltration Co., Ltd. (Hangzhou, China). Triblock copolymer Pluronic F127 (Mw = 12600 EO_106_-PO_70_-EO_106_) was supplied from Sigma-Aldrich (St. Louis, MO, USA). Other chemicals used for the synthesis of the resol precursor were purchased from Sinopharm Chemical Regent Co., Ltd. (Shanghai, China). 

### 3.2. Preparation of Ionic Liquid Confined in Mesoporous Polymer Membrane

The porous Al_2_O_3_ support was obtained using a method similar to that described in the literature [35]. In a typical process, 0.45 g alumina powders were pressed into 18 mm disk at 8 MPa. Then, a porous Al_2_O_3_ support was obtained after sintering at 1400 °C for 10 h. The resol precursor was obtained using a soft-template method described in the literature [24,25]. Typically, phenol (0.61 g) was melted at around 40 °C in a beaker. Under stirring, 0.13 g NaOH aqueous solution (20 wt %) was added slowly. Then, 1.05 g formalin (37 wt %) was added dropwise, and the reaction mixture was stirred at 70 wt % for 60 min. After cooling the mixture to room temperature, the pH of the reaction mixture was adjusted to neutral (7.0) using 0.6 M HCl solution. Water was removed under vacuum below 45 °C. The above resol precursor was dissolved in ethanol, forming 20 wt % ethanolic solution, which was further added to the ethanol solution of pluronic F127 under stirring with the molar composition of phenol/formaldehyde/NaOH/F127 of 1:2:0.1:0.003–0.008. Then, 0.2 mL of the above homogeneous resol solution was spread on the surface of the Al_2_O_3_ support, followed by the solvent evaporation in a petri dish for 3 h, and subsequently in the oven at 100 °C for 24 h. It is necessary to point out that the drying processes probably have an important influence on the structure of the mesoporous membrane and thus its performance. Here, we employed the common drying procedure. After further treating in flowing argon in a quartz tube furnace at 350 °C for 5 h with a ramping rate of 1 °C/min, the mesoporous polymer membrane on the Al_2_O_3_ support was obtained. The coating, drying, and calcination processes were repeated three times to repair the possible defects. 

The supported ionic liquid membrane was prepared as follows. First, 0.15 g [emim][BF_4_] was dropped onto the surface of the mesoporous membrane, then introduced into the mesopores of the phenolic resin on the Al_2_O_3_ support under a vacuum of 0.03 MPa, forming ionic liquid confined in the mesoporous polymer membrane on the Al_2_O_3_ support. The excess ionic liquid was carefully wiped off using a filter paper.

### 3.3. Gas Permeation Measurements

**Figure 8:**
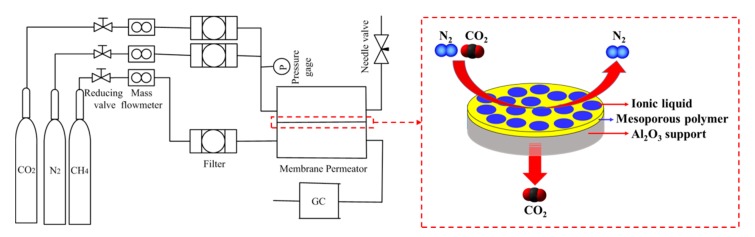
Schematic diagram of membrane permeation unit (GC: gas chromatograph, P: Pressure).

Figure 8 shows the schematic flowcharts of the membrane permeation measurement. The system consists of a feed cell and a permeate cell separated by an SILM, which was sealed by O-rings. CO_2_ and N_2_ were fed with a flow rate of 6 mL/min, while CH_4_ was always fed to the permeate side as a sweep gas with a flow rate of 1 mL/min under atmospheric pressure. CH_4_, CO_2_, and N_2_ flow rates were controlled by mass-flow controllers (Bronkhorst, Ruurlo, The Netherlands). Trans-membrane pressure was monitored using a pressure gauge at the feed side. The gases in the permeation side included the sweep gas and the permeated gas. The flow rate of the gas at the outlet in the permeation side was measured by a bubble flow meter, while gas composition at the outlet was determined by a gas chromatograph (Agilent 7890, Agilent Technology, Santa Clara, CA, USA) equipped with a thermal conductivity detector (TCD).

The selectivity of CO_2_/N_2_ is defined as the ratio of permeance of CO_2_ to that of N_2_. The ideal selectivity of CO_2_/N_2_ can be calculated by Equation (1):

(1) $$\alpha =\frac{J_{{\mathrm{CO}}_{2}}\times y_{{\mathrm{CO}}_{2}}}{J_{N_{2}}\times y_{N_{2}}}$$

where $J_{{\mathrm{CO}}_{2}}$ and $J_{N_{2}}$ are the volumetric flow rates of the permeate side, which are composed of membrane permeance and sweep gas and are determined by a bubble flow meter with the feed of CO_2_ and N_2_, respectively. *y_i_* is the fraction of the component *i* in the permeate side determined by the gas chromatograph (*i* = CO_2_ and N_2_).

The actual selectivity of CO_2_/N_2_ is computed by Equation (2):

(2) $$\alpha =\frac{y_{{\mathrm{CO}}_{2}}}{y_{N_{2}}}\times \frac{x_{N_{2}}}{x_{{\mathrm{CO}}_{2}}}$$

where *x_i_* is the fraction of the component *i* in the feed side determined by the gas chromatograph (*i* = CO_2_ and N_2_). 

### 3.4. Characterization

The Fourier-transform infrared (FTIR) spectra of the Al_2_O_3_ support, the mesoporous polymer membrane on the Al_2_O_3_ support, [emim][BF_4_] confined in the mesoporous polymer membrane on the Al_2_O_3_ support, and pure ionic liquid [emim][BF_4_] were obtained on a Nicolet iN10 spectrophotometer (Thermo fisher Scientific, Waltham, MA, USA). A KBr disc was used as a specimen substrate to collect the FTIR spectrum of pure ionic liquid [emim][BF_4_]. Scanning electron microscopy (SEM) images and elemental mapping spectra were carried out on Hitachi S-4800 microscopy (Hitachi, Tokyo, Japan). Transmission electron microscopy (TEM) images were performed on an H-7650 microscope (Hitachi, Tokyo, Japan) under an accelerate voltage of 100 kV.

## 4. Conclusions

In summary, a novel supported ionic liquid membrane with good stability was developed by confining ionic liquid [emim][BF_4_] in a mesoporous polymer membrane on an Al_2_O_3_ support. The supported ionic liquid membrane exhibits a high ideal CO_2_/N_2_ selectivity of 40 based on the permeances of pure CO_2_ and pure N_2_, and an actual selectivity of around 25 in a mixed CO_2_/N_2_ gas at a trans-membrane pressure difference of 2.5 bar. In contrast to ionic liquid [emim][BF_4_] confined in a macroporous PES membrane, the ionic liquid confined in a mesoporous polymer membrane with a uniform pore size of 7 nm exhibits good stability, which was steadily operated for more than 40 h under a trans-membrane pressure difference of 1.5 bar in a mixed CO_2_/N_2_ gas.
